# The Impact of Coconut Oil and Epigallocatechin Gallate on the Levels of IL-6, Anxiety and Disability in Multiple Sclerosis Patients

**DOI:** 10.3390/nu12020305

**Published:** 2020-01-23

**Authors:** Jose Luis Platero, María Cuerda-Ballester, Vanessa Ibáñez, David Sancho, María Mar Lopez-Rodríguez, Eraci Drehmer, Jose Enrique de la Rubia Ortí

**Affiliations:** 1 Doctoral Degree School, Catholic University of Valencia San Vicente Martir, 46001 Valencia, Spain; joseluisplateroarmero@gmail.com; 2 Department of Nursing, Catholic University of Valencia San Vicente Martir, 46001 Valencia, Spain; m.cuerda@hotmail.com (M.C.-B.); vanessa.ibanez@ucv.es (V.I.); david.sancho@ucv.es (D.S.); joseenrique.delarubi@ucv.es (J.E.d.l.R.O.); 3 Department of Nursing, Physiotherapy and Medicine, University of Almería, 04120 Almería, Spain; 4 Department of Physical Activity and Sport Sciences, Catholic University of Valencia San Vicente Martir, 46001 Valencia, Spain; eraci.drehmer@ucv.es

**Keywords:** multiple sclerosis, epigallocatechin gallate, coconut oil, interleukin-6, anxiety, disability

## Abstract

Background: Due to the inflammatory nature of multiple sclerosis (MS), interleukin 6 (IL-6) is high in blood levels, and it also increases the levels of anxiety related to functional disability. Epigallocatechin gallate (EGCG) decreases IL-6, which could be enhanced by the anti-inflammatory effect of high ketone bodies after administering coconut oil (both of which are an anxiolytic). Therefore, the aim of this study was to assess the impact of coconut oil and EGCG on the levels of IL-6, anxiety and functional disability in patients with MS. Methods: A pilot study was conducted for four months with 51 MS patients who were randomly divided into an intervention group and a control group. The intervention group received 800 mg of EGCG and 60 mL of coconut oil, and the control group was prescribed a placebo. Both groups followed the same isocaloric Mediterranean diet. State and trait anxiety were determined before and after the study by means of the State-Trait Anxiety Inventory (STAI). In addition, IL-6 in serum was measured using the ELISA technique and functional capacity was determined with the Expanded Disability Status Scale (EDSS) and the body mass index (BMI). Results: State anxiety and functional capacity decreased in the intervention group and IL-6 decreased in both groups. Conclusions: EGCG and coconut oil improve state anxiety and functional capacity. In addition, a decrease in IL-6 is observed in patients with MS, possibly due to the antioxidant capacity of the Mediterranean diet and its impact on improving BMI.

## 1. Introduction

Multiple sclerosis (MS) is a chronic, inflammatory and autoimmune disease of the central nervous system (CNS) that causes a progressive deterioration of the myelin sheath associated with axonal injuries at a neuronal level, leading to functional disability. This neuronal damage is mainly based on high oxidative stress and inflammation in the central nervous system, affecting the permeability of the blood–brain barrier (BBB) and allowing autoreactive T cells, B cells, macrophages and microglia to access and especially damage the white matter in the brain and spinal cord [1,2,3].

Due to the inflammatory nature of MS, several inflammatory markers have been related to the disease, especially interleukin 6 (IL-6) whose levels are significantly high in MS patients [4]. Thus, it has been demonstrated that T cells in MS patients have more IL-6 receptors in peripheral blood than healthy individuals [5]. Due to the fact that it is an inflammatory marker, the amounts increase when the person is overweight or obese, which is determined by means of anthropometric parameters, such as the body mass index (BMI) [6] that is correlated with the individual’s adiposity [7]. Clinically speaking, there is a relation to characteristic aspects of the disease, such as relapses and functional disability [4], and with its pathogenesis [8].

Nonetheless, patients with a higher concentration of IL-6 in serum show higher levels of anxiety [9]. Thus, the most anxious individuals have higher levels of IL-6, regardless of their age or gender [10]. Anxiety has been observed to influence the level of inflammation, increasing the risk of developing inflammatory diseases [11]. The influence of anxiety is due to a transcriptional pattern observed in animal models, in which monocytes are recruited that specifically depend on the increase of IL-6 in serum [12], once anxiety in stressful situations is caused. As a result of this link between the high levels of IL-6 and the perception of anxiety, we can see that anxiety disorders are found amongst the most common psychiatric disorders associated with MS [13]. In addition, anxiety symptoms have an effect on the course of the disease, increasing the levels of fatigue [14] and especially functional disability [15].

In this sense, ketone bodies obtained through hepatic beta-oxidation have shown improvements in inflammatory markers, including lipid markers, glycated haemoglobin (HbA1c) or high-sensitivity C-reactive proteins that are related to an increase in the total antioxidant state in blood [16]. Particularly in MS, the ketone body β-hydroxybutyrate (BHB) activates HCA2 receptors expressed by neuroinflammatory cells, reducing neuroinflammation [17] and achieving a neuroprotective effect [18]. In addition, ketone bodies have decreased the perception of anxiety in animal models with Alzheimer’s disease, where an anxiolytic effect of the ketogenic diet has been observed [19], and in humans of advanced age [20]. Regarding the nutrients capable of providing higher levels of ketone bodies in blood, those with high levels of medium-chain triglycerides (MCTs) stand out, with coconut oil possibly being the food with the highest amount of MCTs, due to the high percentages of medium-chain fatty acids (MCFAs), such as lauric acid, palmitic acid, stearic acid, myristic acid and oleic acid [21]. These acids are absorbed intact and do not suffer degradation and reesterification processes [22].

On the other hand, epigallocatechin gallate (EGCG) is a polyphenolic catechin with a high antioxidant and anti-inflammatory activity [23,24]. EGCG’s protective effect allows it to be especially efficient in autoimmune diseases, such as MS, as it promotes the repression of autoreactive T cell proliferation, a reduced production of proinflammatory cytokines, a decrease in Th1 and Th17, as well as an increase in the regulatory T cell populations in lymphoid tissues and the CNS [25]. It also takes the spotlight due to its capacity to penetrate the blood–brain barrier [26] and to accumulate inside the mitochondria of the neurons, decreasing apoptosis due to the high oxidative stress of the disease [27].

Finally, EGCG is also related to an emotional improvement, especially in terms of anxiety, as the activity of GABA receptors is modulated [28]. This would explain the decrease in anxiety after administration in CNS diseases, such as schizophrenia [29]. Therefore, the aim of this study was to assess the impact of coconut oil and EGCG on the levels of IL-6 and anxiety related to functional disability in MS patients.

## 2. Materials and Methods

A prospective, mixed and experimental pilot study was conducted by means of a clinical trial.

### 2.1. Subjects

The population sample was obtained from the main state-wide MS associations who were previously informed on the characteristics of the study. Sixty-seven people were interested in voluntarily taking part in the study. The following selection criteria were applied: patients over 18 years of age diagnosed with MS at least 6 months prior and treated with glatiramer acetate and interferon beta. On the other hand, the exclusion criteria included: pregnant or breastfeeding women, patients with tracheotomy, stoma or with short bowel syndrome, patients with dementia, evidence of alcohol or drug abuse, with myocardial infarction, heart failure, cardia dysrhythmia, symptoms of angina or other heart conditions, patients with kidney conditions with creatinine levels two times higher than normal markers, patients with elevated liver markers three times higher than normal or with chronic liver disease, patients with metabolic or endocrine diseases such as hyperthyroidism or diabetes, patients with acromegaly, patients with polycystic ovary syndrome or MS patients who were included in other researches with experimental drugs or treatment.

### 2.2. Statistical Analysis

Statistical analysis was performed with the SPSS v.23 (IBM Corporation, Armonk, NY, USA) tool. The first step aimed to estimate the distribution of the variables investigated through statistical methods to assess normality, including the Kolmogorov–Smirnov Test. This analysis demonstrated the non-normal distribution of all the scale variables studied. Therefore, the U de Mann–Whitney test and the Wilcoxon signed-rank test were used to assess the inter-group and pre-post differences, respectively. Categorical data were analysed with a chi-square test. A *p*-value below 0.05 was considered significant. Data are presented as mean ± standard deviation, or the number of patients and percentage.

### 2.3. Procedure

Once the final population sample was obtained, the patients received further information on the study, as well as the set objectives and the tests and analyses that would be carried out. They were also provided with instructions to not change the prescribed diet for each case (depending on whether they were in the control group or intervention group), as well as to take the capsules on a daily basis at the scheduled times over the 4-month duration of the intervention. In order to verify the patients complied with the treatment, members of the team made weekly telephone calls (on Monday mornings) to each and every patient. They were asked about any doubts or incidences regarding the diet, with the aim of ensuring the caloric intake was followed, or whether they had had any issue with the capsules (such as an intolerance or side effect). These calls were carried out over the 4 months of the duration of the intervention. No general issues or problems with the diet or capsules were registered.

### 2.4. Intervention

Once the selection criteria had been applied, a final sample of 51 MS patients was obtained. They were randomly divided into the intervention and control group. The intervention group received an isocaloric diet for 4 months (adapted to the individual characteristics of each patient and divided into 5 meals a day: breakfast, mid-morning snack, lunch, afternoon snack and dinner) enriched with 60 mL of extra virgin coconut oil divided into 2 equal intakes (30 mL for breakfast and 30 mL for lunch), and supplemented with 800 mg of EGCG administered in two capsules of 400 mg to be taken twice a day (once capsule in the morning and another in the afternoon).

On the other hand, the control group followed the same isocaloric diet as the intervention group for the same 4 months, as well as administering placebo (capsules containing microcrystalline cellulose, matching in size and colour). They followed the same instructions as the intervention group. The base diet followed by both groups included the following percentage distribution of the 3 main macronutrients with respect to the total caloric value: 20% proteins, 40% carbohydrates and 40% Mediterranean lipids.

This diet was based on the Mediterranean-type food pattern, and was characterised for being balanced, varied and with sufficient calories, by providing adequate food proportions divided into 5 daily intakes. The consumption of proteins with a high biological value of animal origin such as fresh fish, eggs and dairy products (milk, yoghurt and cheese) were highlighted, to the detriment of meat and meat products. In addition, plant protein was provided based on a combination of cereals and pulses. In terms of carbohydrates, they were complex and rich in fibre, provided from rice, cereals, wholegrain bread, pulses, tubers, vegetables and fruit. Regarding lipids, there was a predomination of monounsaturated fatty acids from extra virgin olive oil and nuts rich in omega-3 and omega-6, thus minimising the intake of saturated fatty acids. It is also important to highlight the high level of antioxidants in the diet, especially polyphenols estimated from the work carried out by Manach et al. [30] of 758.85 mg per day, per kg of various fresh foods containing them.

### 2.5. Measurements

The following measurements were taken before and after the 4-month intervention, in the same conditions and at the same time. In the specific case of the scales, they were carried out by the same neurologist assigned to each patient before the study.

#### 2.5.1. State-Trait Anxiety Inventory (STAI)

This scale, used in clinical settings to diagnose anxiety and distinguish it from symptoms of depression [31], was published by Spielberger et al. [32] and validated for the Spanish population [33]. It is commonly used to obtain a significant measurement of state anxiety and trait anxiety. State anxiety refers to how the subject feels in a specific moment, while trait anxiety is described as how the individual normally feels. The inventory is made up of 40 questions, 20 about trait anxiety and 20 about state anxiety. All of the questions are given a 4-point frequency scale. The higher the points, the higher the perceived anxiety.

#### 2.5.2. Expanded Disability Status Scale (EDSS)

This scale is used to assess functional disability in multiple sclerosis patients [34]. The scale is an ordinal scale based on a neurological examination of the eight functional systems (pyramidal, cerebellar, brainstem, mental, sensory, visual, bowel and bladder), alongside an assessment on walking capacity, which, as a result, provides a disability index between 0 and 10, 0 being understood as having normal health and 10 death by MS.

#### 2.5.3. Blood Test and IL-6 Analysis

Blood tests were carried out in the peripheral vein (antecubital vein) at 11 a.m. on an empty stomach. The blood samples were collected in BD Vacutainer Plus serum blood collection tubes (ref. 367815). Once the test was finished, the samples were left at room temperature for 30 min to coagulate. The coagulated part was separated by centrifuging the samples at 4000 rounds/min for 10 min in a refrigerated centrifuge. After centrifuging, the supernatant liquid (blood serum) was transferred to 0.5 mL aliquots, which were then frozen and stored at −80 °C. Finally, the concentration of IL-6 in serum was determined after 24 h had passed. In order to do so, the aliquots were thawed and the ELISA technique (R&D Systems) was used.

#### 2.5.4. Body Mass Index (BMI)

The body mass index is calculated as weight (kg)/height (m^2^). Body weight was measured while the patient was wearing light clothes, no shoes and using a medical scale with a 0.5 kg precision. Height was measured with each patient standing up and using a medical tape measure with a 1 cm precision.

### 2.6. Ethical Concerns

The study was conducted in accordance with the Helsinki Declaration [35], prior approval of the protocol by the Human Research Committee of the University of Valencia of the Experimental Research Ethics Committee (procedure number H1512345043343). In addition, patients included in the study signed a consent form after being informed on the procedures and nature of the study.

## 3. Results

This study analysed a sample of 51 MS patients, divided into an intervention group and a control group, whose socio-demographic and clinical characteristics are shown in Table 1.

After the 4-month intervention, the changes in the analysed variables of the study are shown in Table 2 as the mean and standard deviation. Regarding the intervention group, a significant decrease in serum concentration was produced for IL-6: patients’ anxiety for this group also varied after the diet, significantly decreasing state anxiety, and a significant improvement is shown in functional capacity. Regarding the control group, a significant decrease in the levels of IL-6 in blood is also observed. However, there is no improvement in the level of state or trait anxiety. No change was observed in terms of functional capacity. It is also noteworthy that there was a significant decrease in BMI in both groups.

## 4. Discussion

Among the most representative inflammation biomarkers that activate the immune system caused by stress is IL-6 [36]. This explains that in an immune disease like MS with high oxidative stress, its levels are increased [4]. In this sense, both EGCG and ketone bodies obtained from following ketogenic diets show anti-inflammatory effects [37,38], thus decreasing the levels of IL-6 in blood: EGCG as it negatively regulates its gene expression [39], and ketone bodies block NMDA receptors [40]. After conducting our study, indeed the results prove a decrease in IL-6 levels in the group that received both EGCG and coconut oil. Nonetheless, this significant improvement has also been observed in the control group. This could be explained by the high levels of antioxidants in the Mediterranean diet followed by both groups. The Mediterranean diet is characterised by containing soluble or low molecular weight antioxidants, such as vitamin C and vitamin E, phenolic compounds and carotenoids, and other macromolecular antioxidants that are polymeric phenolic compounds or polyphenols and carotenoids linked to macromolecules of plant foods, which contribute to the antioxidant capacity of the diet [41]; including polyphenols that are greatly contained in fruit and drinks such as tea, red wine and coffee, or in vegetables, pulses and cereals in the Mediterranean diet [30]. In addition, a significant improvement in BMI is observed in both groups that, according to other studies, is positively correlated with IL-6 in blood [42,43], which could explain the decrease in the proinflammatory cytokine in both the control group and the intervention group.

In terms of assessing the psychological variables, although both depression and anxiety disorders are the most frequent nosological entities in MS, the repercussions of symptoms of depression in this pathology have been studied more, and fewer studies assess the impact of anxiety. However, the relation with functional performance and cognitive capacity for MS is only established with levels of anxiety and not with levels of depression [44]. On the other hand, two kinds of anxiety can be differentiated: state anxiety and trait anxiety. According to Spielberger (1972) [45], state anxiety entails an immediate “emotional state” that can be modified in time, while trait anxiety is considered to be relatively stable. In this sense, hyperactivity of the limbic system has been observed in anxiety disorders that could be a result of: a decrease in the inhibitory neurotransmitters (γ-aminobutyric acid, GABA), an increase in the excitatory neurotransmitters based on the action of glutamate or a combination of both [46].

Regarding our intervention, the association of EGCG and ketone bodies causes a significant improvement in state anxiety. This fact seems to be explained through both their action mechanisms. On the one hand, EGCG increases inhibition mediated through the GABA neurotransmitter, which would generate a similar activity to that caused by some medications, such as benzodiazepines [47,48,49], also decreasing levels of occasional anxiety and causing an anxiolytic effect, as observed after administering it in MS patients [28]. Completing this EGCG activity and based on mechanisms that trigger state anxiety, the action mechanism that ketone bodies have that improves anxiousness [19,20] is based on the inhibition of the activation of NMDA ionotropic glutamate receptors. These receptors have an essential role, not only on a cognitive level [50], but also regarding the presence of anxiety [51]. On the other hand, anxiety is felt both on a physiological level, as well as on a cognitive and a mental level [52]; therefore, symptoms have a negative effect on the cognitive function of those who suffer from anxiety. Anxiety symptoms are especially associated with a lower cognitive function [53] in MS, showing a similar pattern to that observed in people with other immune-mediated inflammatory diseases (IMID) and in individuals without an IMID [54]. Therefore, anxiety in MS patients is related to functional disability and constitutes an indicator of the level of this disability [15]. Our results are in line with this idea, as the patients from the intervention group whose levels of anxiety decrease also show a significant improvement in functional capacity. Nonetheless, we must remember that regarding the two types of anxiety, it is state anxiety that significantly improved, therefore it seems that functionality is more directly related to this kind of anxiety and not trait anxiety. These findings would be supported by the results obtained by other authors, who observed how state anxiety (consisting of a temporary emotional state or condition of the human organism that can vary over time and whose intensity can vary), yet not trait anxiety, predicts the performance in the executive function index. Thus, we can conclude that improving state anxiety improves patients’ functionality [44].

However, these results need to be confirmed as this study is somewhat limited. These limitations include a small sample and the lack of intervention groups to study the single contribution of EGCG and coconut oil to improve different variables. Therefore, future research should involve a larger sample leading to a more complex statistical analysis, as well as studying different groups of patients using EGCG, coconut oil and Mediterranean diet, separately.

## 5. Conclusions

Once the intervention with MS patients was carried out, we observed a decrease in state anxiety, and possibly as a result of this, an improvement in the functional capacity of these patients. However, these improvements do not seem to be a direct consequence of the decrease of IL-6 levels in blood, as this can be observed in both groups (intervention group and control group). This could be due to the antioxidant capacity of the Mediterranean diet that all participants of the study followed, as well as the improvements that this diet shows in terms of BMI.

## Figures and Tables

**Table 1:** Socio-demographic and clinical characteristics of the population of the study.

Measure		Group						*p*
		CG *N* = 24		IG *N* = 27		Total *N* = 51		
		Count	%	Count	%	Count	%	
MS type	Relapsing-Remitting	17	70.8%	20	74.1%	37	72.5%	0.796
	Secondary-Progressive	7	29.2%	7	25.9%	14	27.5%	
Gender	Man	10	41.7%	5	18.5%	15	29.4%	0.070
	Woman	14	58.3%	22	81.5%	36	70.6%	
**Measure**		**Median**	**Range**	**Median**	**Range**	**Median**	**Range**	***p***
Age (years)		50.50	45.00	45.00	48.00	47.00	48.00	0.119
Time since diagnosis (years)		13.50	35.00	9.00	35.00	12.00	37.00	0.156
IL-6 pre-test (pg/mL)		2.69	9.98	2.18	16.27	2.54	16.27	0.481
IL-6 post-test (pg/mL)		0.94	3.89	0.84	9.80	0.86	9.86	0.380
STAI state pre-test		19.50	29.00	23.00	38.00	22.00	38.00	0.720
STAI state post-test		20.00	33.00	17.00	34.00	19.00	36.00	0.242
STAI trait pre-test		24.50	42.00	32.00	49.00	28.00	51.00	0.223
STAI trait post-test		23.50	54.00	28.00	54.00	24.00	57.00	0.741
EDSS pre-test		3.75	6.50	3.00	6.50	3.50	6.50	0.435
EDSS post-test		3.75	6.50	3.00	5.50	3.50	6.50	0.351
BMI pre-test (kg/m^2^)		24.61	23.17	23.43	19.09	24.24	23.17	0.992
BMI post-test (kg/m^2^)		23.54	21.74	23.49	18.05	23.54	21.74	0.946

Z: U de Mann Whitney; CG: control group; IG: intervention group; MS: multiple sclerosis; EDSS: Expanded Disability Status Scale; IL-6: interleukin 6; STAI: State-Trait Anxiety Inventory; BMI: body mass index; SD: standard deviation.

**Table 2:** Differences between the study variables after intervention.

**IG**			
**Measure**	**Mean**	**SD**	***p***
EDSS pre-test	3.37	2.03	0.047 *
EDSS post-test	3.28	1.87	
IL-6 pre-test (pg/mL)	3.66	4.10	0.000 *
IL-6 post-test (pg/mL)	1.31	2.09	
STAI state pre-test	22.26	8.99	0.049 *
STAI state post-test	17.67	10.62	
STAI trait pre-test	30.11	11.67	0.134
STAI trait post-test	26.89	11.97	
BMI pre-test (kg/m^2^)	25.92	5.29	0.002 *
BMI post-test (kg/m^2^)	25.16	4.94	
**CG**			
**Measure**	**Mean**	**SD**	***p***
EDSS pre-test	3.80	2.00	0.655
EDSS post-test	3.86	2.08	
IL-6 pre-test (pg/mL)	3.67	2.94	0.001 *
IL-6 post-test (pg/mL)	1.37	1.15	
STAI state pre-test	21.71	9.00	0.833
STAI state post-test	21.48	9.39	
STAI trait pre-test	27.04	12.21	0.457
STAI trait post-test	25.83	11.93	
BMI pre-test (kg/m^2^)	25.87	6.10	0.012 *
BMI post-test (kg/m^2^)	25.36	5.85	

EDSS: Expanded Disability Status Scale; IL-6: interleukin 6 (mean value of normal IL-6, 1.4 pg/mL); STAI: State-Trait Anxiety Inventory; *: statistically significant differences *p* < 0.05; Z: Wilcoxon signed-rank test; BMI: body mass index; SD: standard deviation.
